# Free‐breathing 2D radial cine MRI with respiratory auto‐calibrated motion correction (RAMCO)

**DOI:** 10.1002/mrm.29499

**Published:** 2022-11-08

**Authors:** Guruprasad Krishnamoorthy, Joao Tourais, Jouke Smink, Marcel Breeuwer, Marc Kouwenhoven

**Affiliations:** ^1^ Department MR R&D–Clinical Science Best The Netherlands; ^2^ Department of Biomedical Engineering Eindhoven University of Technology Eindhoven The Netherlands

**Keywords:** Cardiac MRI, Free‐breathing Cine, Non‐cartesian acquisition, respiratroy motion correction

## Abstract

**Purpose:**

To develop a free‐breathing (FB) 2D radial balanced steady‐state free precession cine cardiac MRI method with 100% respiratory gating efficiency using respiratory auto‐calibrated motion correction (RAMCO) based on a motion‐sensing camera.

**Methods:**

The signal from a respiratory motion‐sensing camera was recorded during a FB retrospectively electrocardiogram triggered 2D radial balanced steady‐state free precession acquisition using pseudo–tiny‐golden‐angle ordering. With RAMCO, for each acquisition the respiratory signal was retrospectively auto‐calibrated by applying different linear translations, using the resulting in‐plane image sharpness as a criterium. The auto‐calibration determines the optimal magnitude of the linear translations for each of the in‐plane directions to minimize motion blurring caused by bulk respiratory motion. Additionally, motion‐weighted density compensation was applied during radial gridding to minimize through‐plane and non‐bulk motion blurring. Left ventricular functional parameters and sharpness scores of FB radial cine were compared with and without RAMCO, and additionally with conventional breath‐hold Cartesian cine on 9 volunteers.

**Results:**

FB radial cine with RAMCO had similar sharpness scores as conventional breath‐hold Cartesian cine and the left ventricular functional parameters agreed. For FB radial cine, RAMCO reduced respiratory motion artifacts with a statistically significant difference in sharpness scores (*P* < 0.05) compared to reconstructions without motion correction.

**Conclusion:**

2D radial cine imaging with RAMCO allows evaluation of left ventricular functional parameters in FB with 100% respiratory efficiency. It eliminates the need for breath‐holds, which is especially valuable for patients with no or impaired breath‐holding capacity. Validation of the proposed method on patients is warranted.

## 1 INTRODUCTION

Cardiac cine MRI is clinically accepted as a gold standard to assess left‐ventricular (LV) volume and functional assessment.^1^, ^2^ Functional parameters that are derived from this approach, such as end‐systolic volume, end‐diastolic volume, ejection fraction, and stroke volume, can be used to diagnose heart disease. An external electrocardiogram is typically used to synchronize the acquisition to freeze the heart's contractile motion in each of the reconstructed cardiac phases. Balanced steady‐state free precession (bSSFP) is usually the preferred acquisition sequence for cine MRI.^3^ It offers a high signal‐to‐noise ratio with good image contrast between blood and myocardium.^4^

A 2D Cartesian bSSFP sequence with retrospective cardiac gating is commonly used in routine clinical practice. Retrospective gating allows coverage of the entire cardiac cycle, and 2D slice selective excitation provides enhances the bSSFP blood‐myocardium contrast due to inflow of unsaturated blood. To cover the whole left ventricle, multiple sequential 2D slices are acquired. These are acquired in breath‐holds (BH) to minimize respiratory motion artifacts, usually with 1 or 2 slices per BH, typically in 8 to 12 s per BH. Performing multiple BHs requires patient cooperation, which can be challenging for severely ill or uncooperative patients.^5^ With Cartesian imaging, no or poor BHs can lead to respiratory ghosting artifacts.^6^ Also, inconsistencies between different BHs can result in slice misalignment, potentially leading to deviations in functional assessment.^7^ Therefore, it would be of clinical interest to acquire the cine images in free‐breathing (FB) while minimizing respiratory motion blurring and ghosting artifacts.

Several FB alternatives to the standard 2D Cartesian bSSFP sequence have been proposed. Prospective respiratory gating/triggering techniques acquire data only in a limited (e.g., expiratory) respiratory window,^8^, ^9^, ^10^, ^11^, ^12^, ^13^ which can result in a typical respiratory gating efficiency of 50% or less, and the total scan time and the resulting image quality depend on the patient's respiratory pattern. Other approaches reduce acquisition times with sparse reconstruction techniques such that the scan time per slice is faster than the respiratory cycle to reduce motion artifacts in a FB acquisition.^14^ These approaches have fixed acquisition times irrespective of the breathing pattern because no respiratory gating is applied, but patient cooperation is required to perform shallow breathing. In retrospective techniques,^15^, ^16^, ^17^ the data is acquired without any prospective respiratory gating, and the artifacts caused by the respiratory motion are retrospectively mitigated in the reconstruction. They do not require patient cooperation and have fixed acquisition times. In particular, techniques with 100% respiratory gating efficiency^15^, ^16^ are interesting because they allow FB without increased scan times. These techniques were based on radial sequences because of the robustness to motion^18^ and used sparsity‐based reconstruction techniques to maintain shorter scan times while minimizing respiratory motion artifacts. However, these approaches have long reconstruction times, which can limit its application in clinical settings.

This study proposes a new FB cine approach with 100% respiratory gating efficiency based on a multi‐slice 2D radial bSSFP cine sequence with a fully automated retrospective respiratory motion‐corrected reconstruction called *respiratory auto‐calibrated motion correction* (RAMCO). We use tiny‐golden‐angle (tGA),^22^ radial k‐space ordering to reduce artifacts related to eddy currents associated with rotating consecutive radial projections and to disperse the motion uniformly throughout the k‐space for increased robustness to motion artifacts in a FB acquisition. The proposed method was implemented on the scanner platform with inline reconstruction of images for easier clinical workflow. The proposed method was evaluated on 9 volunteers, and the LV functional assessment parameters and image sharpness were compared with a conventional BH^car^ technique.

## 2 METHODS

### 2.1 Acquisition

The proposed FB radial (FB^rad^) cine sequence with retrospective cardiac gating was implemented on a 1.5 Tesla scanner (Ingenia Ambition X, Philips Healthcare, Best, The Netherlands). We used pseudo–tiny‐golden‐angle (pseudo‐tGA) radial ordering, an extension of tGA.^19^, ^20^, ^21^ With pseudo‐tGA, a set of uniformly spaced radial projections is temporally re‐ordered such that the angle between subsequent projections is as close as possible to tGA.^22^ The standard tGA acquisition scheme generates a non‐equidistant angular spacing between adjacent projections. In contrast, pseudo‐tGA generates a uniform distribution of radial projections in k‐space, reducing aliasing artifacts compared to tGA. Supporting Information Figure S1 shows the radial trajectory difference between the standard tGA and the pseudo‐tGA used in this study. During the acquisition of each radial projection in FB^rad^, the respiratory signal from a motion‐sensing camera (VitalEye, Philips Healthcare, Best, The Netherlands) was recorded to perform retrospective Emotion correction.

In vivo experiments were performed in 9 volunteers—7 males and 2 females, aged 55.4 ± 14.3 years—under a protocol approved by the ethics committee with written informed consent. In all 9 volunteers, scout images along the 3 orthogonal planes of the thoracic cavity were obtained using a non‐electrocardiogram–gated BH bSSFP sequence. Based on these scout scans, a series of 10 to 12 contiguous short‐axis slices covering the entire LV from apex to the base were planned and acquired using standard BH Cartesian (BH^cart^). A local shim volume was specified tightly covering the heart to improve field homogeneity and thus minimize off‐resonance effects. An identical slice prescription was used with the proposed FB^rad^. In 1 of the volunteers, an additional FB acquisition was performed with linear radial ordering to compare the effect of radial ordering on respiratory motion artifacts. Volunteers were not given any special instructions for the FB technique. Imaging parameters for both BH^cart^ and FB^rad^ are detailed in Table 1. Sixteen‐element anterior and 12‐element posterior phased array coils were used for signal reception. The signals from individual coil elements were combined,^27^ and to correct the signal uniformity a constant level appearance algorithm was applied.^28^ Inline reconstruction was performed on the scanner using the proposed RAMCO motion‐correction algorithms and gridding implemented in Recon 2.0 (Philips Healthcare, Best, The Netherlands) with a reconstruction time of ∼30 s per dataset for the proposed FB^rad^ method.

**TABLE 1:** Acquisition parameters for the standard Cartesian BH cine method and the proposed FB radial cine method

Parameters	BH Cartesian cine (BH^cart^)	FB radial cine (FB^rad^)
Trajectory	Cartesian	Radial
Scan technique	bSSFP	
Acceleration	2 (SENSE)	1 (None)
Partial Fourier	0.7 (phase‐encode direction)	1 (None)
No. of Acquired profiles	∼51	∼188
TE/TR (msec), FA	∼1.2/2.4, 60^0^	∼1.2/2.5, 60^0^
Bandwidth	1085 Hz/Pixel	
Acquired temporal resolution	40–45 msec (25 heart phases)	
Acquired/Recon voxel size (mm)	2.5 × 2.5/1.25 × 1.25	
FOV (mm)	300 × 300	
Slice thickness/gap (mm)	10/0	
Total slices	12	
Scan time/slice	∼3.0 s	∼12.0 s
Respiratory compensation	2 slices/breath hold	Free breathing
Total scan time	∼2.3 min	∼2.5 min

Abbreviation: BH, breath‐holds; BH^car^, BH Cartesian cine; bSSFP, balanced steady‐state free precession; FB, free‐breathing; FB^rad^, FB radial cine.

### 2.2 Motion‐corrected reconstruction

**FIGURE 1:**
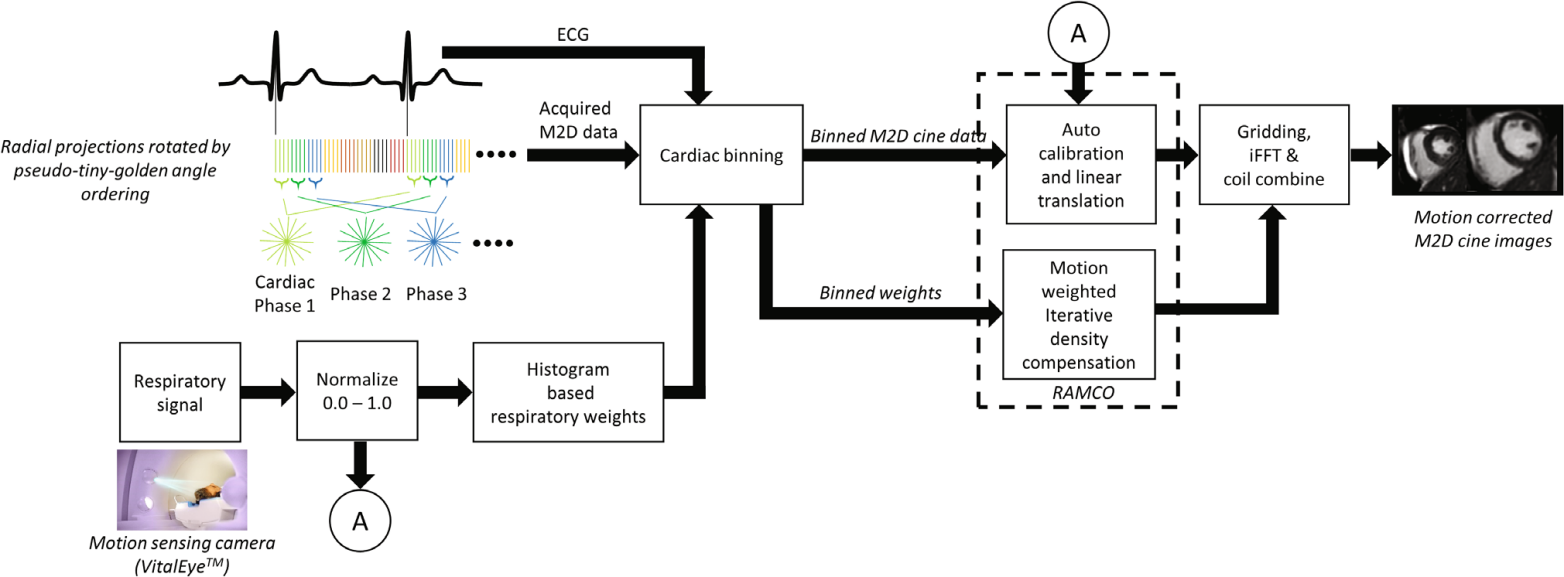
Schematic diagram of the proposed respiratory motion‐corrected FB radial cine reconstruction framework using RAMCO. Respiratory motion artifacts caused by in‐plane bulk motion were minimized with linear translations based on the auto‐calibrated respiratory signal from a motion sensing camera. Through‐plane motion and residual in‐plane motion were minimized with respiratory motion‐weighted density compensation applied in the gridding. FB, free‐breathing; RAMCO, respiratory auto‐calibrated motion correction.

The radial projections were binned into multiple cardiac phases according to the electrocardiogram to resolve the contractile motion of the heart. The following processing steps were applied to minimize artifacts caused by the respiratory motion of the heart: (1) Respiratory signals with arbitrary units obtained using the motion‐sensing camera were retrospectively calibrated for each subject to obtain actual in‐plane translation of the heart with physical units, and linear phase shifts were applied in k‐space based on the calibrated signal to minimize bulk in‐plane respiratory motion of the heart; and (2) motion‐weighted density compensation was applied during gridding to minimize the residual in‐plane respiratory motion (rotation, contraction, and expansion) and the through‐plane motion (translation, rotation, contraction, and expansion) based on the histogram of the calibrated signal. The proposed reconstruction framework is outlined in Figure 1. Each of the steps for respiratory motion correction is explained in detail in the following sections.

#### 2.2.1 Respiratory auto‐calibrated motion correction (RAMCO)

**FIGURE 2:**
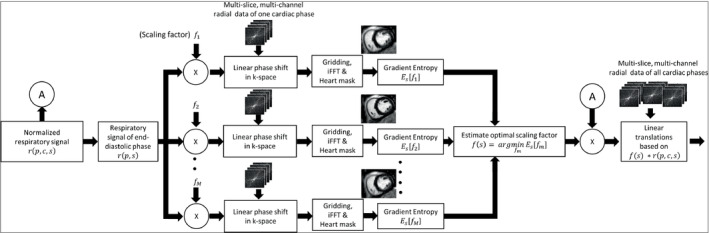
Overview of the auto‐calibration and linear translation components of RAMCO. By minimizing $E_{s}$, an optimal scaling factor for each slice in 1 cardiac phase is identified. This scaling factors (f(s)) are then used to scale the respiratory signal and in turn used to perform linear translation of data corresponding to all cardiac phases. Es, motion‐artifact metric

The process of performing respiratory motion correction in k‐space based on a respiratory signal from the motion‐sensing camera is described in this section. A schematic diagram of this process is outlined in Figure 2. The respiratory signal r(n,s) obtained using the motion‐sensing camera was normalized such that the minimum value is 0, and the maximum value is 1, with n=1,2,….N radial projections acquired per slice for s=1,2,….S slices. Based on a histogram of the normalized respiratory signal, a respiratory sample $r\left(n^{\mathrm{ref}},s^{\mathrm{ref}}\right)$ is identified such that there is the least difference between the value of the sample and the value at the peak of the histogram. The value of this reference sample was subtracted from the values of all the respiratory samples $r\left(n,s\right)=r\left(n,s\right)-r\left(n^{\mathrm{ref}},s^{\mathrm{ref}}\right)$ so that the samples corresponding to the most common respiratory motion state have values close to 0. This subtraction was done to avoid shifting the heart images due to the linear translations. The normalized respiratory signal was then binned into multiple cardiac phases as r(p,c,s) with p=1,2,…P radial projections per cardiac phase, and c=1,2,..C cardiac phases and s=1,2..S slices. Supporting Information Figure S2 shows various 1D plots of a respiratory signal obtained using the motion‐sensing camera before and after the normalization process. From the C cardiac phases, the respiratory signal and the complex k‐space data of only 1 cardiac phase was chosen for the auto‐calibration process to reduce the computation time substantially. Auto‐calibration was done only on 1 cardiac phase, assuming that the correlation between the respiratory signal from the motion‐sensing camera and the physical, respiratory motion of the heart is independent of its contractile motion state. For simplicity, the respiratory signal corresponding to the chosen cardiac phase is denoted henceforth as r(p,s) by excluding the cardiac phase index.

Given that, $K_{x}$ and $K_{y}$ are the in‐plane k‐space coordinates in directions x and y of a radial projection at measurement sample index k,ce is the surface coil element index, and θ is the angle of through‐plane rotation of the acquired 2D slice with respect to the magnet's *Z*‐axis. $f_{m}$ is a scaling factor in the superoinferior (SI) direction with m=1,2,…M scaling factors. For a given radial projection, linear translation was applied in SI direction on the complex radial data R(k,p,ce,s) as follows,

(1a) $$\overset{ˇ}{R}\left(k,p,s,\mathrm{ce},m\right)=R\left(k,p,\mathrm{ce},s\right)*e^{-i2\pi *f_{m}*r\left(p,s\right)*K_{\mathrm{SI}}\left(k\right)}$$

(1b) $$K_{\mathrm{SI}}\left(k\right)=K_{y}\left(k\right)*\cos\left(\theta \right)-K_{x}\left(k\right)*\sin\left(\theta \right),$$

where $\overset{ˇ}{R}\left(k,p,s,\mathrm{ce},m\right)$ is the translated radial data based on $f_{m}$. The translated radial data were gridded onto a Cartesian grid, and inverse Fourier transform was applied to obtain I(s,ce,m). Where I is image corresponding to slice s, coil‐element ce and scaling factor. The resulting images corresponding to multiple coil elements were combined using the sum‐of‐squares algorithm to obtain M multi‐slice images I(s,m).

After performing the linear translations in SI direction, a scaling factor ($f_{m}$) that yielded the best motion‐corrected images of the heart from I(s,m) was identified. For an automatic and objective evaluation, a motion‐artifact metric based on gradient entropy was used.^23^ The gradient entropy decreases when the motion blurring is minimized in an image. The following equations are used to estimate gradient entropy (E) of a motion‐corrected image,^24^

(2a) $$E=-\sum_{\mathrm{ij}}p_{\mathrm{ij}}\log_{2}\left[p_{\mathrm{ij}}\right]$$

(2b) $$p_{\mathrm{ij}}=\frac{h_{\mathrm{ij}}}{\sum_{\mathrm{ij}}\left(h_{\mathrm{ij}}\right)}$$

(2c) $$h_{\mathrm{ij}}=\sqrt{{\left(I\left(i+1,j\right)-I\left(i,j\right)\right)}^{2}+{\left(I\left(i,j+1\right)-I\left(i,j\right)\right)}^{2}},$$

where the pixel value of image I at index i,j is denoted as I(i,j). Estimating this metric globally in a motion‐corrected image is not optimal because the organs moving with the respiration (e.g., liver, heart) and the stationary organs (e.g., subcutaneous fat, neck) might be affected quite differently in the image due to the linear translations. Hence, a spatial mask was used to limit this motion‐artifact metric computation to the heart region of interest (ROI) in each slice. This mask was automatically generated using the geometry of shim volume that was set during the scan planning to minimize off‐resonance effects. An optimal scaling factor ($f_{\mathrm{SI}}\left(s\right)$) that yielded the best motion correction of the heart in SI direction for a given slice was identified as follows,

(3) $$f_{\mathrm{SI}}\left(s\right)=\arg\min_{f_{m}}E_{s}\left[f_{m}\right],$$

where $E_{s}\left[f_{m}\right]$ is the gradient entropy score for a given slice that is motion‐corrected based on a given scaling factor $\left[f_{m}\right]$. A similar approach was followed to estimate an optimal scaling factor in anteroposterior (AP) direction $f_{\mathrm{AP}}\left(s\right)$ but with the following differences: (1) R(k,p,s) was first motion‐corrected by applying a linear phase shift in SI direction based on $f_{\mathrm{SI}}\left(s\right)$ according to the formulation in Equation (1) to obtain $R^{\mathrm{SI}}\left(k,p,s\right)$; this motion‐corrected radial data was then used to estimate $f_{\mathrm{AP}}\left(s\right)$; and (2) linear translations in AP direction were done as follows.

(4a) $$\hat{R}\left(k,p,s,\mathrm{ce},m\right)=R^{\mathrm{SI}}\left(k,p,\mathrm{ce},s\right)*e^{-i2\pi *f_{m}^{\prime }*r\left(p,s\right)*K_{\mathrm{AP}}\left(k\right)}$$

(4b) $$K_{\mathrm{AP}}\left(k\right)=K_{y}\left(k\right)*\sin\left(\theta \right)-K_{x}\left(k\right)*\cos\left(\theta \right),$$

where $\hat{R}\left(k,p,s,\mathrm{ce},m\right)$ is the radial data translated in AP direction based on a scaling factor $f_{m}^{\prime }$ in AP direction with m=1,2,…M scaling factors. After obtaining the optimal scaling factors in SI and AP directions, respiratory motion correction is performed in the k‐space data of all the cardiac phases as follows,

(5) $$\begin{array}{cc} \hat{R}\left(k,p,c,\mathrm{ce},s\right) & =R\left(k,p,c,\mathrm{ce},s\right)\\ & *e^{-i2\pi *r\left(p,c,s\right)*\left(f_{\mathrm{SI}}\left(s\right)*K_{\mathrm{SI}}\left(k\right)+f_{\mathrm{AP}}\left(s\right)*K_{\mathrm{AP}}\left(k\right)\right)}. \end{array}$$

In the current implementation, the scaling factors for SI ($f_{m}$) and AP ($f_{m}^{\prime }$) directions were chosen such that the full range of possible translational motion of the heart due to respiration was considered.^25^ The minimum and maximum values for the scaling factors were set as 0 to 8.0 and −3.0 to 3.0 for SI and AP directions, respectively, with M = 25. For an acquisition voxel size of 2.5 mm, these scaling factors translate to bulk respiratory motion of 0 to 20.0 mm and −7.5 to +7.5 mm in SI and AP directions, respectively.

Although the linear translations can minimize the artifacts caused by the in‐plane bulk respiratory motion, residual non‐translational in‐plane and through‐plane motions can lead to local blurring in the reconstructed image. This local blurring was minimized by applying a density compensation function for each radial projection according to the current respiratory position. First, a motion weight for each radial projection was obtained according to a histogram of the normalized respiratory signal from the motion‐sensing camera. These weights are denoted as w(n,s), where n=1,2..N radial projections acquired and s=1,2..S slices. Then, w(n,s) is binned to multiple cardiac phases based on the electrocardiogram signal, similar to the binning of radial projections to obtain w(p,c,s), where p=1,2,…P radial projections per cardiac phase and c=1,2,..C cardiac phases with s=1,2,..S slices. These weights were set as initial weights to the iterative density compensation algorithm.^26^ The resultant mwDCF obtained from this algorithm was used in the gridding of radial data corresponding to each cardiac phase and slice.

#### 2.2.2 Quantitative sharpness analysis

Sharpness scores^29^ were estimated on the blood–myocardial border of the LV in 9 volunteers for the following 3 datasets:

1)
Standard BH^cart^ M2D cine
2)
FB^rad^ cine with no respiratory motion correction (FB^rad^‐NoMC):
3)
FB^rad^ cine with RAMCO (FB^rad^‐MC).

In each dataset, sharpness scores were estimated in the middle 4 slices, with 10 estimates in each slice. An average of these 40 estimates was considered a sharpness score for a given dataset. The differences in sharpness scores between the 3 datasets were evaluated with a 2‐tailed paired Student *t* test, and a *P* value less than 0.05 was considered statistically significant.

#### 2.2.3 Left‐ventricular functional assessment

LV functional measurements, including end‐diastolic volume, end‐systolic volume, ejection fraction, and systolic volume, were computed in the 3 different datasets. These measurements were performed using a commercially available semi‐automatic software package, IntelliSpacePortal 9.0 (Philips Healthcare, Best, The Netherlands). Bland–Altman analysis^30^ and paired 2‐tailed Student *t* test were used to compare the LV functional parameters of FB^rad^ with BH^cart^.

## 3 RESULTS

**FIGURE 3:**
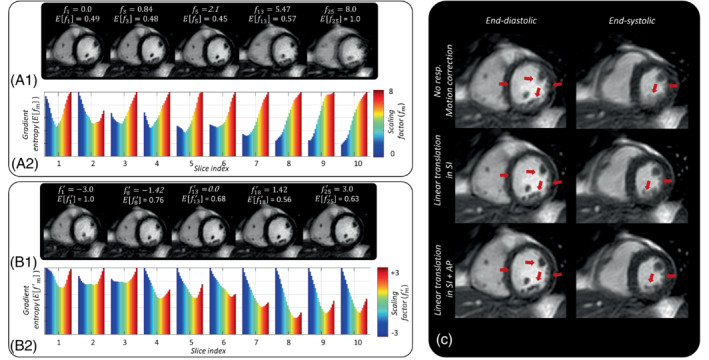
The effect of auto‐calibration and linear translations for a representative volunteer. (A) Images of a midventricular slice in end‐diastolic phase, reconstructed with different linear translations in (A.1) SI and (B.1) AP directions, respectively. The corresponding scaling factors (${f_{m},f}_{m}^{\prime }$) and gradient entropy scores ($E\left[f_{m}\right],E\left[f_{m}^{\prime }\right]$) are displayed on top of the images. Bar plots of gradient entropy score versus scaling factors in different slices are displayed for (A.2) SI and (B.2) AP directions. For each slice, a scaling factor with the lowest gradient entropy score was assumed as an optimal scaling factor to convert unitless respiratory signal to a signal with physical units. (C) End‐diastolic and end‐systolic images from the mid‐ventricular slice with no motion correction, linear translation only in SI direction, and linear translation in SI and AP directions based on the calibrated respiratory signal are shown in the first, second, and third rows, respectively. Note the progressive reduction in motion artifacts AP, anteroposterior, SI, superoinferior.

**FIGURE 4:**
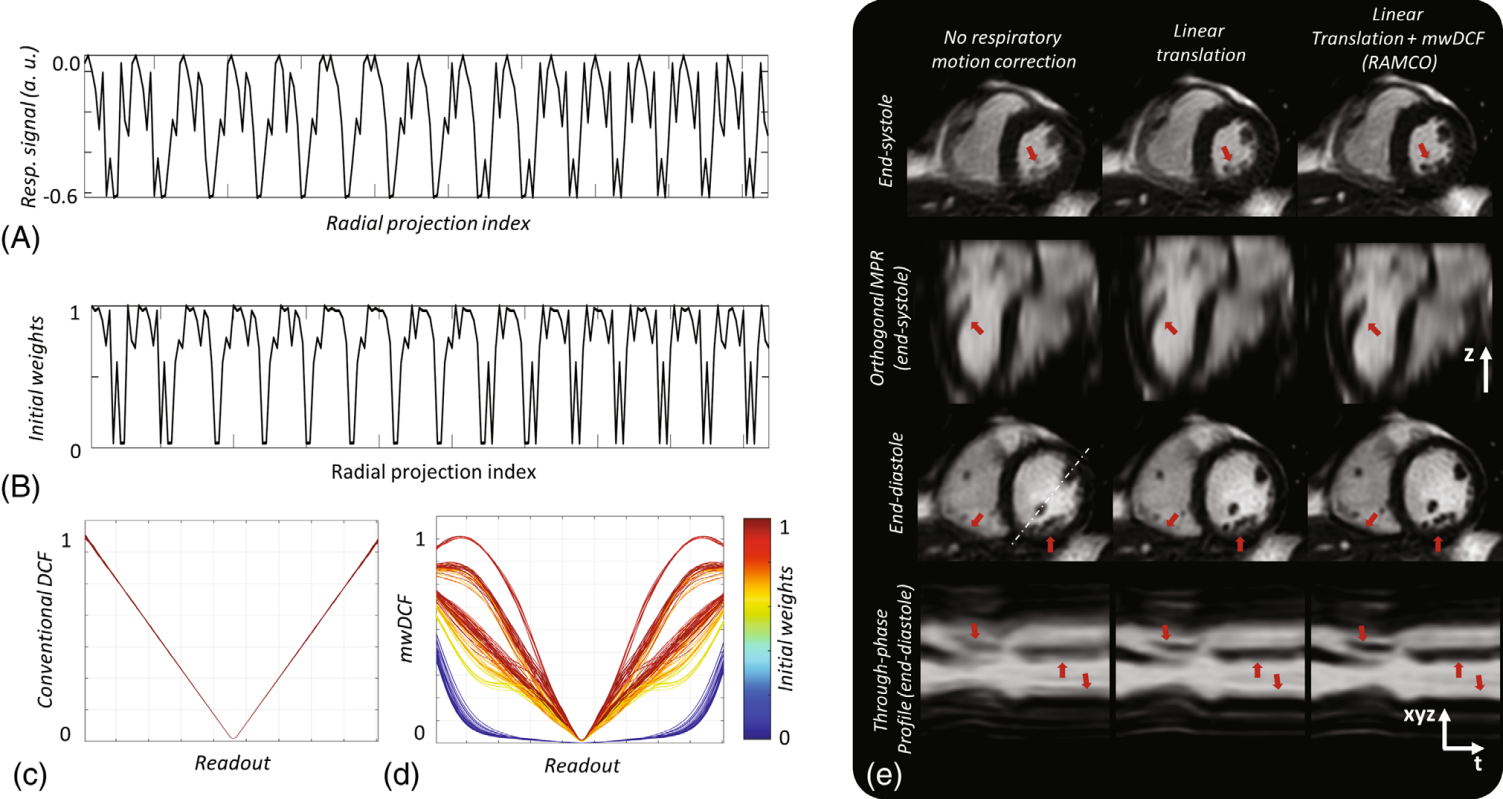
The effect of motion‐weighted density compensation on a representative volunteer. (A) Normalized respiratory signal obtained from the patient sensing camera and (B) the initial weights corresponding to the end‐diastolic images shown in (E). (C) Conventional DCF (D) mwDCF computed using the iterative algorithm with initial weights according to (B). (E) Representative images with no respiratory motion correction, auto‐calibrated linear translations in SI direction, and RAMCO combined with mwDCF. The through‐phase (cardiac) profile shown in the last row of (E) is obtained from an oblique line profile in end‐diastole indicated by a white dotted line through the left ventricle as shown in the third row. Note the progressive reduction in respiratory motion‐artifacts from the left to the right column, indicated by red arrows DCF, density compensation function; *mwDCF*, motion‐weighted density compensation function.

Images with respiratory motion correction using auto‐calibrated linear translations obtained from a representative volunteer are shown in Figure 3. The effect of motion‐weighted density compensation on a representative volunteer is demonstrated in Figure 4. Linear translation minimized motion artifacts by correcting the in‐plane respiratory motion in SI and AP directions. However, some residual blurring was still present. This residual blurring was further minimized by motion‐weighted density compensation function (mwDCF) as indicated by red arrows in Figure 4E.

FB cine images obtained with linear radial ordering and the proposed pseudo‐tGA radial ordering (FB^rad^) are compared in Supporting Information Figure S3. In the images with RAMCO, the pseudo‐tGA had visually sharper images compared to images with linear radial ordering. In the images with no respiratory motion correction, the manifestation of motion artifacts was more localized in the case of linear radial ordering. In contrast, images obtained with pseudo‐tGA had artifacts dispersed throughout the image.

**FIGURE 5:**
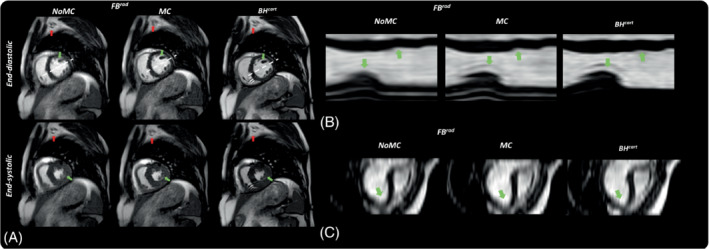
Comparison of cine images obtained with the standard *BH* ^*cart*^ to the proposed *FB* ^*rad*^ *‐NoMC* and with *FB* ^*rad*^ *–MC* (RAMCO) on a volunteer. (A) Representative images in end‐diastole and end‐systole showing the entire FOV. (B) Through‐phase profile of pixel intensities along the white dotted line in the left ventricle shown in (A), and (C) orthogonal 4‐chamber MPR images of the 3 datasets. Note the significant reduction of motion artifacts in the images of *FB* ^*rad*^ *–MC* compared to *FB* ^*rad*^ *–NoMC* . The sharpness of *FB* ^*rad*^ *–MC* (RAMCO) is comparable to that of *BH* ^*cart*^ , as indicated by green arrows. Whereas RAMCO reduced motion artifacts in the heart, it introduced some blurring in regions outside the heart as indicated by red arrows *BH* ^*cart*^ , breath‐holds Cartesian cine; *FB* ^*rad*^ *–MC* , FB radial cine with RAMCO motion correction; *FB* ^*rad*^ *‐NoMC* , FB radial cine without respiratory motion correction.

Cine images obtained using FB^rad^ are visually compared against BH^cart^ in Figure 5. FB^rad^‐MC had comparable image quality as BH^cart^. FB^rad^‐MC significantly reduced the respiratory motion artifacts within the heart visible in FB^rad^‐NoMC. As expected, FB^rad^‐MC introduced slight blurring outside the heart, as indicated by red arrows.

**FIGURE 6:**
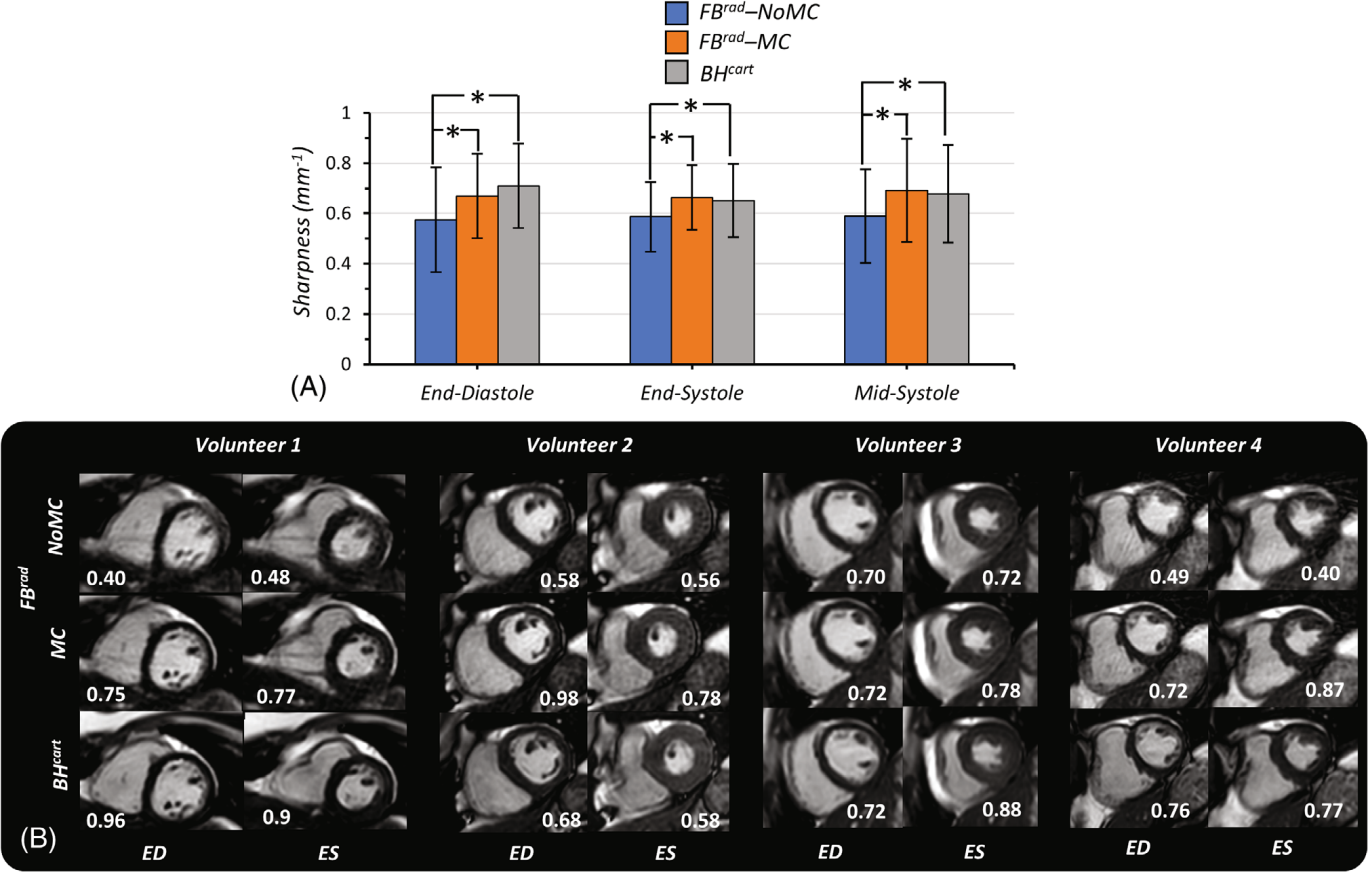
Image sharpness scores. (A) Bar plots of average sharpness scores of the standard *BH* ^*cart*^, *FB* ^*rad*^ *–NoMC*, and with *FB* ^*rad*^ *‐MC* (RAMCO) obtained from 9 volunteers. Statistically significant differences (*P* < 0.05) are indicated by (*). (B) Images obtained with the 3 methods from 4 different volunteers in ED and ES, along with the corresponding sharpness score printed at the bottom of each image ED, end‐diastolic phase; ES, end‐systolic phase.

Average sharpness scores obtained from 9 volunteers in 3 cardiac phases (end‐diastolic, end‐systolic, and mid‐systolic) for the 3 datasets (BH^cart^, FB^rad^‐NoMC, and FB^rad^‐MC) are depicted in Figure 6A. The differences between FB^rad^‐NoMC and BH^cart^ and between FB^rad^‐NoMC and FB^rad^‐MC were statistically significant in all 3 cardiac phases (*P* < 0.05). The differences between FB^rad^‐MC and BH^cart^ in all 3 cardiac phases were not statistically significant (*P* > 0.05). Representative images from 4 volunteers with their corresponding sharpness scores are shown in Figure 6B. The proposed FB^rad^‐MC method corrected most of the motion artifacts visible in FB^rad^‐NoMC reconstruction, achieving visually similar image quality as the standard BH^cart^ acquisition.

**FIGURE 7:**
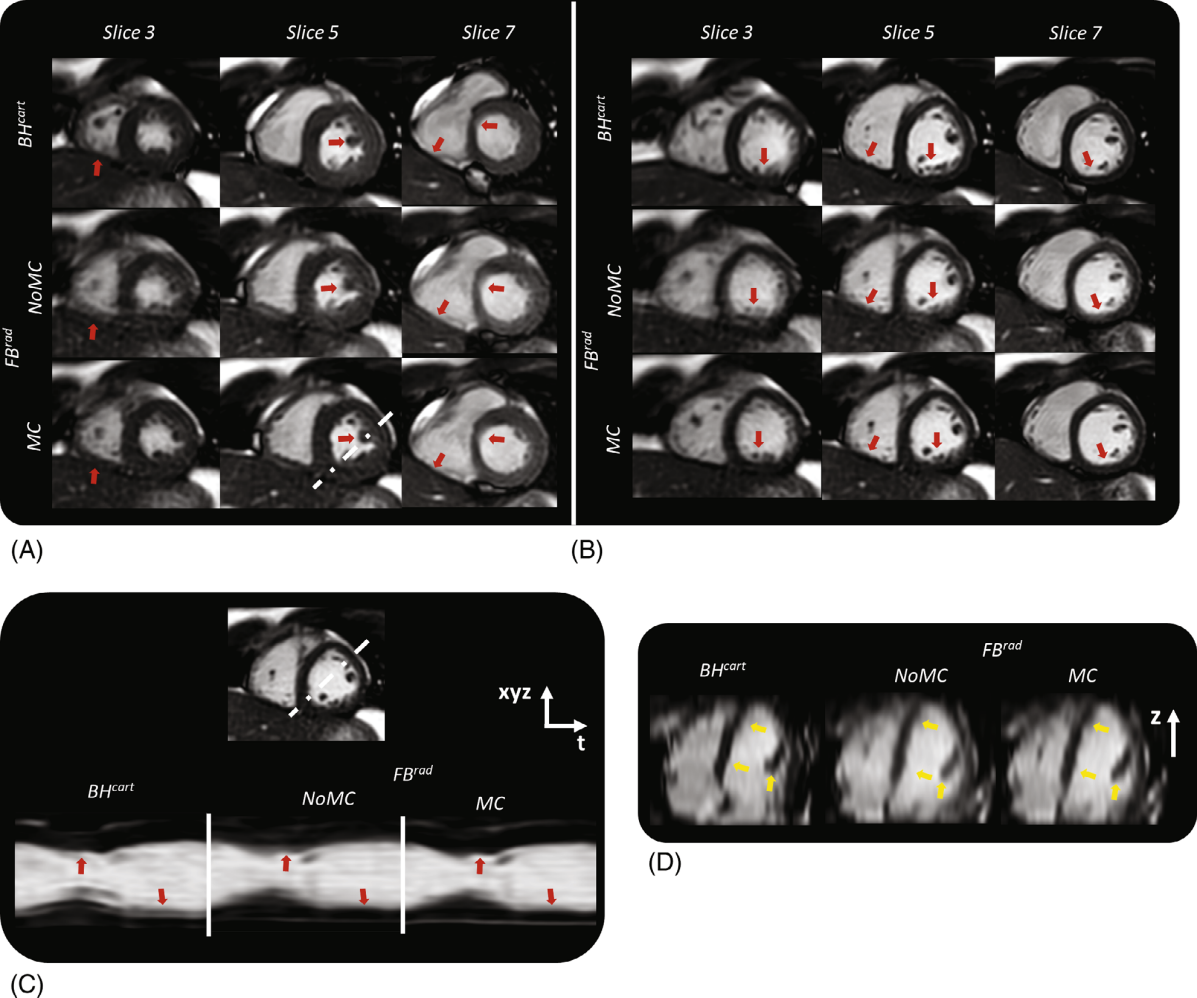
Comparison of the standard *BH* ^*cart*^ to the proposed *FB* ^*rad*^ *‐NoMC* and with *FB* ^*rad*^ *–MC* on a representative volunteer. (A) Representative slices in end‐systolic phase and (B) end‐diastolic phase shown for the 3 methods. (C) Through‐cardiac phase profile along a white dotted line for the 3 methods. *FB* ^*rad*^ *–MC* corrected for most of the local blurring due to respiratory motion and had similar spatial and temporal quality as the *BH* ^*cart*^ as indicated by red arrows. (D) Through‐slice profiles along the oblique axis of the heart shown by white dotted line in (A). Slice mis‐alignments due to inconsistent breath‐holds are clearly visible in *BH* ^*cart*^ that are not present in *FB* ^*rad*^ as indicated by yellow arrows

In Figure 7, the cine images obtained from 1 of the volunteers using BH^cart^, FB^rad^‐NoMC, and FB^rad^‐MC are compared. The local blurring due to the respiratory motion in the FB^rad^ acquisition is visibly minimized using RAMCO, as shown in Figure 7A–C with comparable image quality as BH^cart^. Slice misalignments due to inconsistent BHs were evident in BH^cart^ and avoided in the proposed FB method, as shown in Figure 7D. A video of cine images from 2 representative volunteers obtained with different methods are shown in Supporting Information Video S1.

**FIGURE 8:**
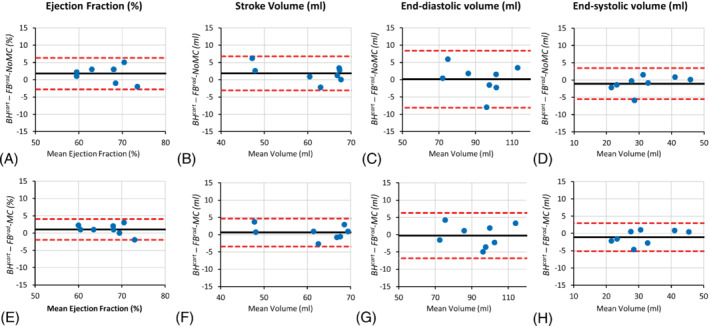
Bland–Altman plots comparing LV functional assessment parameters between (A–D) standard *BH* ^*cart*^ and *FB* ^*rad*^ *‐NoMC*: and between (E–H) *BH* ^*cart*^ and *FB* ^*rad*^ *‐MC*LV, left ventricular.

The mean and SD of LV functional parameter indices measured from the 3 datasets in 8 volunteers are provided in Table 2. We decided not to include this volunteer in the statistical analysis under the assumption that the LV parameters from BH^cart^ are erroneous due to the slice misalignment between multiple BHs. The differences in all LV parameter values between BH^cart^ and FB^rad^‐NoMC and between BH^cart^ and FB^rad^‐MC were not statistically significant (*P* > 0.05). Figure 8 depicts Bland–Altman plots comparing each of these parameters obtained from the 3 datasets. In the volunteer shown in Figure 7, the LV functional parameters obtained using BH^cart^ and FB^rad^ deviated substantially. The values were (parameter = FB^rad^‐NoMC, FB^rad^‐MC, and BH^cart^): end‐diastolic volume (mL) = 80.8, 82.0, and 69; end‐systolic volume (mL) = 30.0, 29.9, and 25.4; ejection fraction (%) = 63, 64, and 63; systolic volume (mL) = 50.8, 52.1, and 43.6.

**TABLE 2:** LV functional parameters in 8 volunteers

Method	EDV (ml)	ESV (ml)	EF (%)	SV (ml)
BH^cart^ (A)	94.04 ± 13.06	31.70 ± 8.19	66.65 ± 5.25	62.18 ± 9.08
FB^rad^‐NoMC (B)	93.90 ± 12.84	32.74 ± 8.49	64.88 ± 6.42	60.31 ± 7.45
FB^rad^‐MC (C)	94.29 ± 12.68	32.78 ± 8.26	65.63 ± 5.50	61.55 ± 7.57
A to B/A (%)	0.08 ± 4.28	−3.43 ± 8.188	2.764 ± 3.479	2.63 ± 3.97
A to B (*p*)	0.93	0.24	0.07	0.07
A to C/A (%)	−0.34 ± 3.46	−3.65 ± 7.14	1.55 ± 2.26	0.64 ± 3.46
A to C (*p*)	0.84	0.19	0.1	0.42

*Note*: EDV, ESV, EF, and SV for 3 different methods: standard *BH* ^*cart*^, *FB* ^*rad*^ *‐NoMC*, and *FB* ^*rad*^ *‐MC*. *P* value from paired 2‐tailed Student *t* test. *P* value less than 0.05 was considered statistically significant.

Abbreviation: EDV, end‐diastolic volume; EF, ejection fraction; ESV, end‐systolic volume; *FB* ^*rad*^ *‐MC*, free‐breathing radial cine with respiratory motion correction; *FB* ^*rad*^ *‐NoMC*, free‐breathing radial cine without respiratory motion correction; KV, left ventricular; SV, stroke volume.

## 4 DISCUSSION

We have presented a motion‐correction method called RAMCO. This method calibrates a unitless respiratory motion signal based on the imaging data to obtain bulk respiratory motion of the ROI in physical units. A linear translation followed by motion‐weighted density compensation is applied to minimize artifacts caused by the respiratory motion of the ROI. We applied this method to FB radial cine imaging with 100% respiratory gating efficiency.

This study used a respiratory signal obtained from the motion‐sensing camera as the motion surrogate for RAMCO. Compared to respiratory bellow, the motion‐sensing camera has the advantage of not requiring any additional setup, which reduces patient preparation time. However, any motion surrogate including respiratory bellow could be used with RAMCO if the signal of the surrogate correlates with the respiratory motion of the heart. If the signal from the surrogate has a poor correlation with the translational motion of the heart, RAMCO will not introduce additional motion blurring because the formulation in Equation (3) will automatically result in a scaling factor equal to 0 ($f_{m}\text{and}/\text{or}f_{m}^{\prime }=0$), resulting in no translation correction.

This study limited the motion‐artifact metric estimation to the heart by applying a mask (i.e., heart is the ROI). We used the geometry of the user‐defined B0 shim volume around the heart for generating heart‐only masks. Alternatively, the mask can also be generated by an automatic heart detection algorithm.^31^ The mask used in this study resulted in a scaling factor that is optimal only for the heart; thus, the linear translation process blurred the tissues that did not move along with the heart, shown by red arrows in Figure 5A. If multiple ROIs are present within the FOV that exhibit different translational motion, multiple motion‐corrected images can be generated for each ROI. These images can be finally combined to form a single image similar to auto‐focus.^24^ RAMCO can be combined with any acquisition technique with arbitrary trajectory using appropriate k‐space coordinates in Equation 1b, 4b. RAMCO can also be modified for other clinical applications with ROIs other than the heart by adapting a mask accordingly.

The mwDCF function visibly minimized the residual motion artifacts seen in the images corrected using linear translations, as seen in Figure 4E. The iterative density compensation algorithms,^26^ used in this study to compute mwDCF converged such that (1) the projections are weighted according to the initial respiratory weights in the central regions of k‐space where there is oversampling, and (2) the projections are weighted uniformly close to the k‐space periphery where there is no oversampling. As a result, residual motion artifacts (caused by in‐plane affine, rotation, and through‐plane motion of the heart) were minimized without increased aliasing artifacts, shown in Figure 4D.

In most volunteer scans that we performed for the short‐axis cine, the slice orientation was oblique axial, which means there was already a substantial respiratory motion in the through‐plane. In cases where the most dominant motion is a through‐plane (true axial) motion for 2D acquisitions, the in‐plane linear translation part of RAMCO may not substantially minimize artifacts. The mwDCF may still minimize respiratory motion artifacts, and we believe applying mwDCF should be better than no motion correction in these cases. However, further investigation is required to evaluate the performance of mwDCF for minimizing the artifacts caused by the most dominant through‐plane motion. Also, further investigation is warranted to test the performance of RAMCO in other cine views such as long axis, 2‐chamber, and 4‐chamber. However, this case is only applicable to 2D acquisitions. When the range of motion of the ROI is within the FOV in 3D acquisitions, the calibration and linear translation can be extended to correct respiratory motion in all 3 directions in combination with mwDCF.

In our current implementation, we performed the subject‐specific retrospective auto‐calibration (RAMCO) using the radial data of only 1 of the cardiac phases (end‐diastole). We used the estimated scaling factors to correct for respiratory motion in the images of all the cardiac phases. The entire reconstruction process was implemented in C++ and took full advantage of multiple cores in the CPU. Per dataset (all slices and phases), RAMCO took ∼20 s, and the rest of the reconstruction steps, namely, gridding and coil combination, took ∼10 s on a system with 10‐core CPU with 128 GB of RAM.

Free breathing radial with RAMCO (FB^rad^‐MC) had slightly higher sharpness scores in the end‐systolic and mid‐systolic phases and a slightly lower score in end‐diastolic cardiac phases than the Cartesian BH, as shown in Figure 6. These differences were not statistically significant though. Given the fact that the radial acquisition was acquired in free breathing with 100% respiratory efficiency, the similar sharpness scores show the effectiveness of RAMCO. As expected, the free breathing radial without RAMCO had significantly lower sharpness scores. As shown in Figure 6B, Cine images obtained with the radial sequence had an overall increased level of pseudo‐random noise‐like and streaking artifacts compared to BH Cartesian. This increased level of artifacts is likely due to respiratory motion combined with 100% gating efficiency, aliasing, flow, and eddy currents.

The LV functional parameters obtained using FB^rad^‐MC agreed with the parameters obtained using BH^cart^, with no statistically significant differences. Although the LV parameters obtained with FB^rad^‐MC matched more closely with BH^cart^ than FB^rad^‐NoMC, there was no statistically significant difference between the LV parameters of BH^cart^ and FB^rad^‐NoMC either. Based on the preliminary study, we ascribe these results to the robustness to respiratory motion artifacts of the radial acquisition trajectory used in this study. However, more volunteers and patients need to be evaluated to confirm this effect.

The proposed FB radial method has several strengths. Slice mismatch due to inconsistent BHs can be avoided in the FB method, as shown in Figure 7. The FB radial technique allows acquiring cine images with a high temporal resolution at the cost of longer acquisition times, which could broaden the clinical applications of cine sequences, such as assessing diastolic function. RAMCO can be used with 100% respiratory gating efficiency, but it could potentially also be combined with respiratory triggering or gating with a lower respiratory gating efficiency to reduce residual motion artifacts at the cost of longer scan times. The proposed method is also compatible with prospective arrhythmia rejection, allowing the acquisition even for patients with irregular heartbeats.

The current implementation of the radial cine acquisition did not use any acceleration techniques. For this reason, even with 100% respiratory efficiency, the total net scan time for the proposed free breathing radial method was similar to that of the BH Cartesian method, which used SENSE acceleration but was limited by recovery times between the different BHs. The actual radial data acquisition time was ∼3× longer than for the Cartesian method. Non‐Cartesian sparse sampling techniques such as compressed sensing can be combined with the proposed method to reduce the acquisition times or apply respiratory gating to reduce residual motion blurring.

## 5 CONCLUSION

A FB multi‐slice 2D radial bSSFP cine acquisition using RAMCO with 100% respiratory efficiency was developed and validated against the standard Cartesian BH cine technique. In our preliminary study, the FB^rad^ method yielded similar sharpness and LV functional assessment as the BH^cart^ cine technique with a similar scan‐time. FB^rad^ cine with RAMCO may be a promising alternative to conventional BH^cart^ cine imaging for evaluating LV function.

## FUNDING INFORMATION

Supported by the European Commission within the Horizon 2020 Framework through the Marie Skłodowska‐Curie Innovative Training Networks (MSCA‐ITN) European Training Networks (ETN), project number 642458

## CONFLICT OF INTEREST

Guruprasad Krishnamoorthy, Jouke Smink, Marc Kouwenhoven, and Marcel Breeuwer are employees of Philips healthcare.

## Supporting information


**FIGURE S1**. Difference in trajectories of tiny‐golden‐angle (tGA) and pseudo‐tiny‐golden‐angle (pseudo‐tGA) radial ordering with the first three acquired projections in red, green and blue, respectively. (A) Radial trajectories with constant azimuthal increments defined by tGA (e.g., ∼23.62°) lead to a slight difference between angles of consecutive radial projections (# and *). (B) With pseudo‐tGA ordering, the tGA angles are rounded such that the end result is a uniform angle between all consecutive radial projections (&). Both figures (A) and (B) contain the same number (21) of radial profiles.
**FIGURE S2**. Normalization and binning of respiratory signal obtained from the motion sensing camera. (A) 1D plot of respiratory signal from motion sensing camera normalized to 0.0–1.0 (B) Histogram of the respiratory signal shown in (A). (C) 1D plot of respiratory signal after subtracting all the values of (A) from the value at the peak (0.89) of the histogram shown in (B). (D) 1D plots of respiratory signals after binning to multiple slices and cardiac phases based on the signal shown in (C). The rows in (D) correspond to the middle three slices and the columns correspond to end‐diastolic and end‐systolic cardiac phases (left and right), respectively.
**FIGURE S3**. The effect of tiny‐golden‐angle for radial. Comparison of free‐breathing radial cine images obtained with linear radial profile ordering (first row) and the proposed pseudo‐tiny‐golden‐angle (ptGA) radial ordering (second row). (A) Images with no respiratory motion correction and (B) with RAMCO motion correction. Note the differences in manifestation of motion artifacts between the two radial orderings within the white dotted circles in (A). With RAMCO, applying ptGA resulted in visually sharper images as highlighted by the red arrow in (B).

**Supporting Information Video S1**. Video of cine images obtained with different methods. Images from two representative volunteers are shown in first and second row, respectively. BH = Breath‐hold, FB = Free breathing, No MoCo = No respiratory motion correction, RAMCO = proposed respiratory motion correction.
