# Supplementary material for: Free‐breathing 2D radial cine MRI with respiratory auto‐calibrated motion correction (RAMCO)
Source: Magn Reson Med. 2022 Nov 8;89(3):977–89. doi: 10.1002/mrm.29499 (PMC10100319; doi:10.1002/mrm.29499)
Supplement: Supplementary file 1 — FIGURE S1. Difference in trajectories of tiny‐golden‐angle (tGA) and pseudo‐tiny‐golden‐angle (pseudo‐tGA) radial ordering with the first three acquired projections in red, green and blue, respectively. (A) Radial trajectories with constant azimuthal increments defined by tGA (e.g., ∼23.62°) lead to a slight difference between angles of consecutive radial projections (# and *). (B) With pseudo‐tGA ordering, the tGA angles are rounded such that the end result is a uniform angle between all consecutive radial projections (&). Both figures (A) and (B) contain the same number (21) of radial profiles. FIGURE S2. Normalization and binning of respiratory signal obtained from the motion sensing camera. (A) 1D plot of respiratory signal from motion sensing camera normalized to 0.0–1.0 (B) Histogram of the respiratory signal shown in (A). (C) 1D plot of respiratory signal after subtracting all the values of (A) from the value at the peak (0.89) of the histogram shown in (B). (D) 1D plots of respiratory signals after binning to multiple slices and cardiac phases based on the signal shown in (C). The rows in (D) correspond to the middle three slices and the columns correspond to end‐diastolic and end‐systolic cardiac phases (left and right), respectively. FIGURE S3. The effect of tiny‐golden‐angle for radial. Comparison of free‐breathing radial cine images obtained with linear radial profile ordering (first row) and the proposed pseudo‐tiny‐golden‐angle (ptGA) radial ordering (second row). (A) Images with no respiratory motion correction and (B) with RAMCO motion correction. Note the differences in manifestation of motion artifacts between the two radial orderings within the white dotted circles in (A). With RAMCO, applying ptGA resulted in visually sharper images as highlighted by the red arrow in (B). [file MRM-89-977-s001.docx]

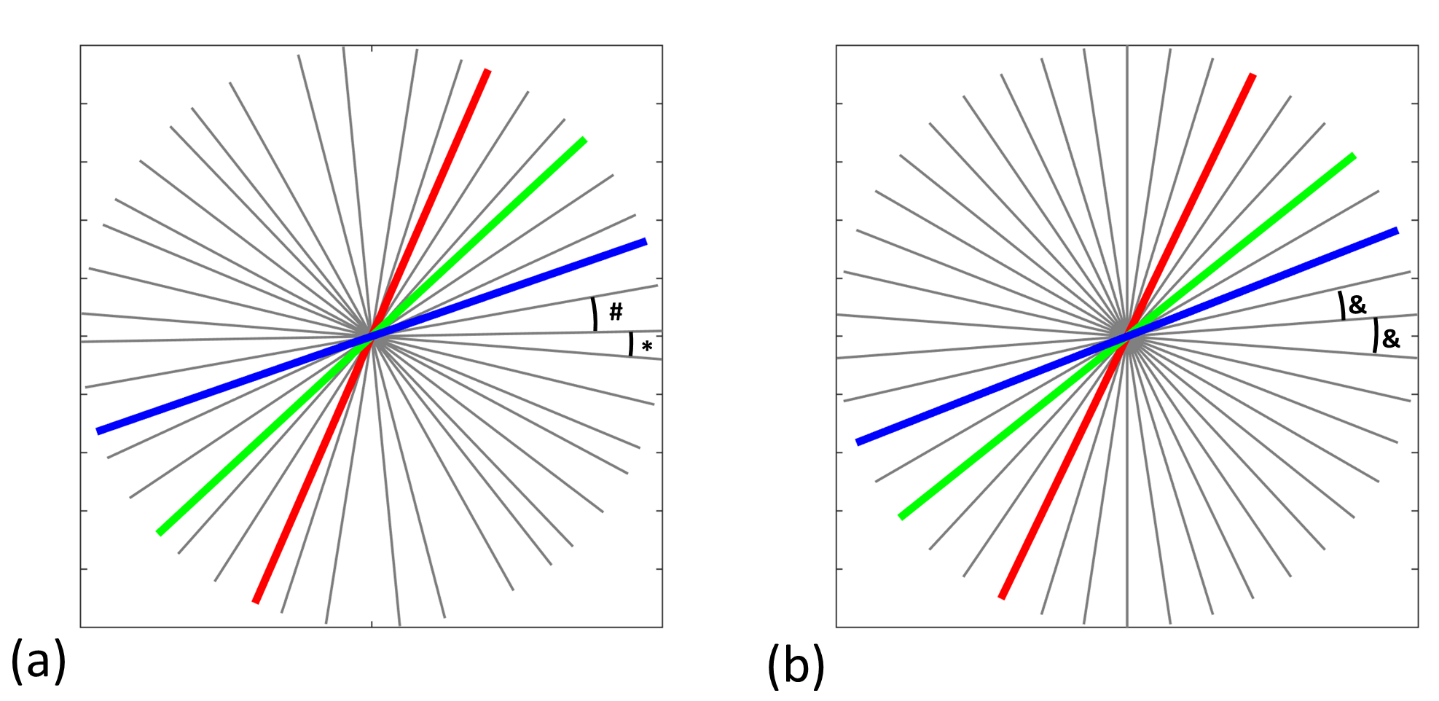


Supporting information figure S1: Difference in trajectories of tiny-golden-angle (tGA) and pseudo-tiny-golden-angle (pseudo-tGA) radial ordering with the first three acquired projections in red, green and blue, respectively. (a) Radial trajectories with constant azimuthal increments defined by tGA (e.g. $\sim23.62^{\circ}$) lead to a slight difference between angles of consecutive radial projections (# and *). (b) With pseudo-tGA ordering, the tGA angles are rounded such that the end result is a uniform angle between all consecutive radial projections (&). Both figures (a) and (b) contain the same number (21) of radial profiles.


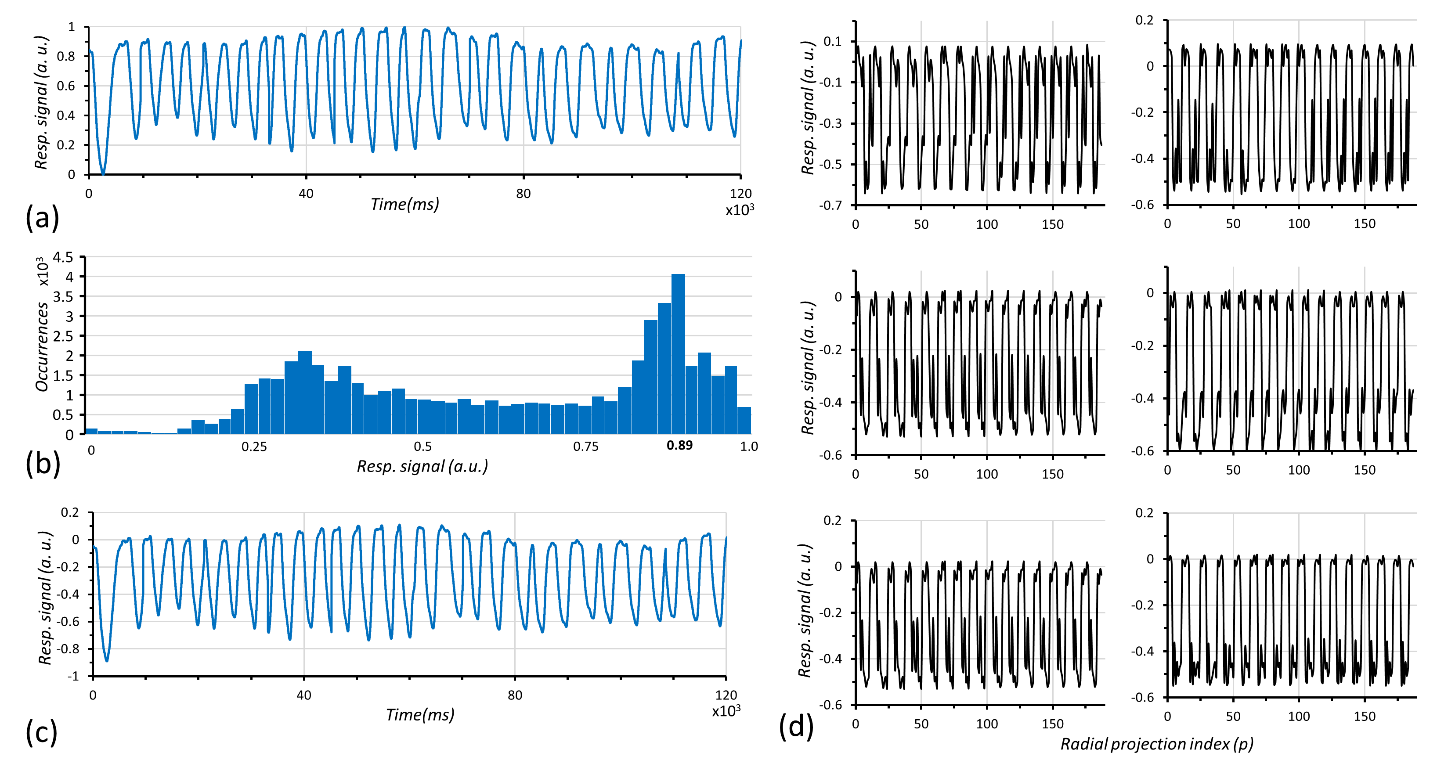


Supporting information figure S2: Normalization and binning of respiratory signal obtained from the motion sensing camera. (a) 1D plot of respiratory signal from motion sensing camera normalized to 0.0-1.0 (b) Histogram of the respiratory signal shown in (a). (c) 1D plot of respiratory signal after subtracting all the values of (a) from the value at the peak (0.89) of the histogram shown in (b). (d) 1D plots of respiratory signals after binning to multiple slices and cardiac phases based on the signal shown in (c). The rows in (d) correspond to the middle three slices and the columns correspond to end-diastolic and end-systolic cardiac phases (left and right), respectively.

Supporting information figure S3: The effect of tiny-golden-angle for radial. Comparison of free-breathing radial cine images obtained with linear radial profile ordering (first row) and the proposed pseudo-tiny-golden-angle (ptGA) radial ordering (second row). (a) Images with no respiratory motion correction and (b) with RAMCO motion correction. Note the differences in manifestation of motion artifacts between the two radial orderings within the white dotted circles in (a). With RAMCO, applying ptGA resulted in visually sharper images as highlighted by the red arrow in (b).


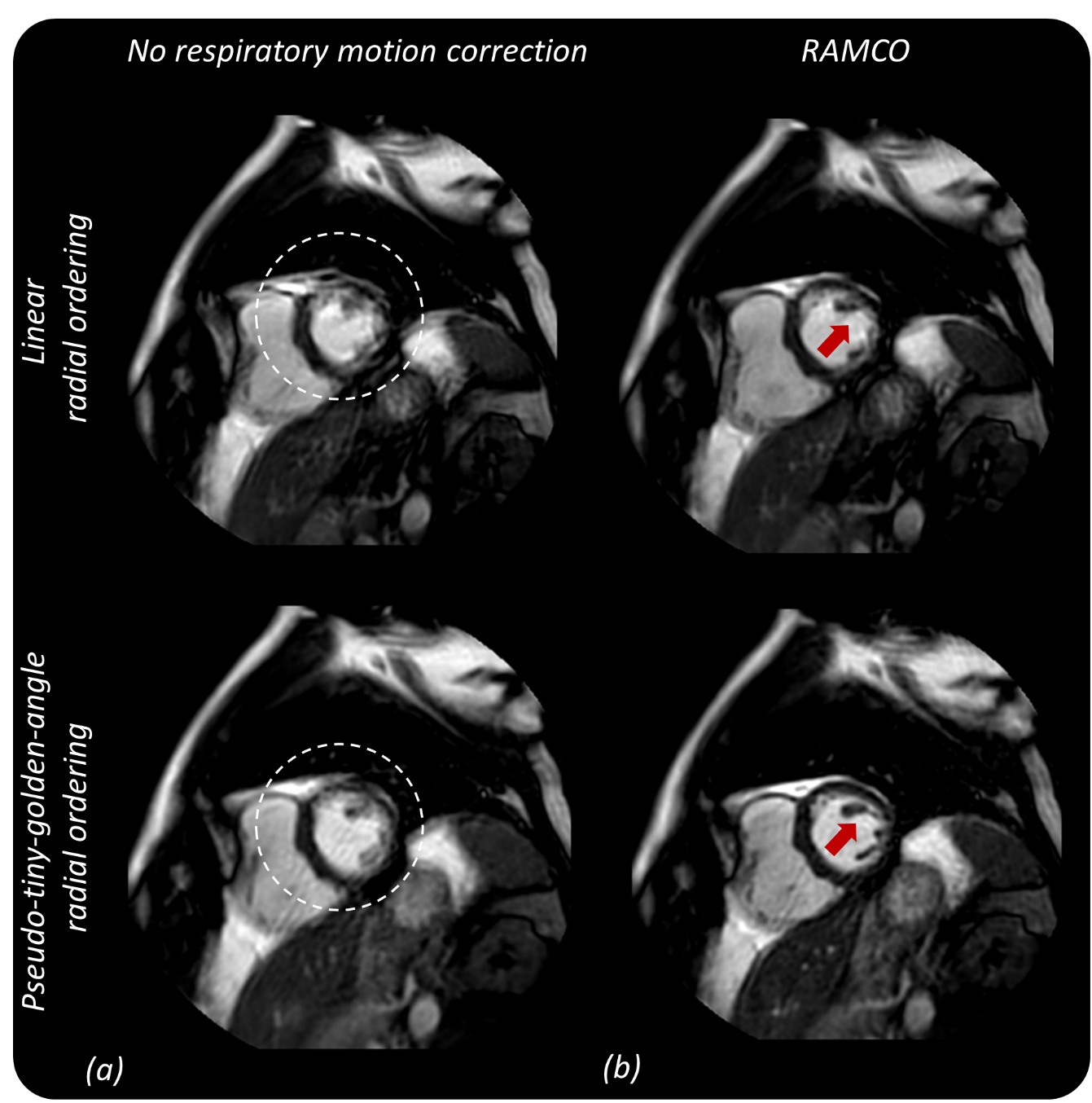


Supporting information video S4: Video of cine images obtained with different methods. Images from two representative volunteers are shown in first and second row, respectively. BH = Breath-hold, FB = Free breathing, No MoCo = No respiratory motion correction, RAMCO = proposed respiratory motion correction.
